# Safe corridor for the implantation of thoracolumbar pedicle screws in growing pigs: A morphometric study

**DOI:** 10.1371/journal.pone.0184857

**Published:** 2017-10-23

**Authors:** Thibaut Cachon, Paul Pillard, Thierry Odent, Claude Carozzo, Eric Viguier

**Affiliations:** 1 Unité ICE (USPS 2016-A104.) Campus Vétérinaire de Lyon-VetAgro-Sup, MARCY L’ETOILE, FRANCE; 2 CHU Tours - Hôpital d'enfants Clocheville- Service de Chirurgie Orthopédique pédiatrique, TOURS, FRANCE; Universidad de Zaragoza, SPAIN

## Abstract

The pig spine is widely used as a large animal model for preclinical research in human medicine to test new spinal implants and surgical procedures. Among them, pedicle screw is one of the most common method of fixation of those implants. However, the pedicle of the porcine vertebra is not as well defined and not as large as the pedicle of the human vertebra. Therefore, the position of the screw should be adapted to the pig and not merely transposed based on the literature on humans. The purpose of this study is to determine the characteristics of the optimum implantation corridors for pedicle screws in the thoracolumbar spine of piglets of different ages using computed tomography (CT) and to determine the size and length of these corridors in pigs of different ages. CT scans from five groups of age: 6, 10, 14, 18, and 26 weeks were reviewed. For each thoracolumbar vertebrae, the pedicle width, pedicle axis length, and the pedicle angle was measured for the left and right pedicle. A total of 326 thoracic vertebrae and 126 lumbar vertebrae were included in the study. Pedicles are statistically larger but not longer for the lumbar vertebrae. An important variation of the pedicle angle is observed along the spine. In all pigs, an abrupt modification of the pedicle angle between T10 and T11 was observed, which corresponds to the level of the anticlinal vertebra which is the vertebra for which the spinous process is nearly perpendicular to the vertebral body. In conclusion, this study provides a quantitative database of pedicle screw implantation corridors in pigs of different ages. When using pedicle screws in experimental studies in pigs, these results should be considered for selecting the most suitable implants for the study but also to ensure a correct and safer screw position. Improving study procedures may limit postoperative complications and pain, thereby limiting the use of live animals.

## Introduction

Scoliosis is a complex three-dimensional deformity of the spine affecting the orientation, position and morphology of the vertebrae in 3 dimensions. Adolescent idiopathic scoliosis is the most common type of scoliosis [1]. For many years, the standard surgical treatment when bracing was not sufficient has been spinal fusion to achieve an acute and stable correction of the spine. However, several issues, such as reduced mobility, reduced thoracic cage volume, reduced stature and the crankshaft phenomenon, are observed with these types of treatments in young patients. Therefore, to avoid these complications, surgical techniques and implants are being developed to achieve scoliosis correction without fusion of the spine. The goal of these fusionless surgeries is to control the deformity in all three dimensions while preserving the growth of the spine [2, 3]. Because there are no reported spontaneous animal models of scoliosis, scoliosis animal models have been developed and reported in the literature [4]. The induction of scoliosis-like deformities has been described in small species such as rabbits, chickens and rats as well as in large animals such as pigs, cows, goats and sheep. Numerous prenatal, systemic, or local surgical procedures are needed to create experimental scoliosis within these animal species. Because of their size, large-animal models are more suitable than small-animal models for the development of new corrective devices. Among the large-animal models, domestic pigs appear to be one of the most suitable species for preclinical testing [5–8] because their spines have a similar size and shape as the human spine and because of their growth potential and early weaning. Finally, domestic pigs are readily available, inexpensive, easy to handle and well accepted as an ethical animal model [4, 9, 10].

In most cases, spinal implant fixation [6–8] is performed with pedicular screws. Pedicular screws are the strongest means of fixation of the growing rod in pigs [11]. As in humans, the size and position of the screw should be adapted to the vertebra to avoid violation of the vertebral canal during screw insertion and to limit screw pull out during the postoperative period. However, the pedicle of the porcine vertebra is not as well defined and not as large as the pedicle of the human vertebra. Therefore, the position of the screw should be adapted to the pig and not merely transposed based on the literature on humans.

The purpose of this study is to determine the characteristics of the optimum implantation corridors for pedicular screws in the thoracolumbar spine of piglets of different ages using computed tomography (CT) and to determine the size and length of these corridors in pigs of different ages.

## Materials and methods

This study was approved by the ethical committee of VetAgro-Sup.

CT scans on immature French Landrace pigs, performed for other experimental studies unrelated to this study, were reviewed. All pigs were from the same breeders and free of vertebral pathology. Spinal CT scans of pigs from T1 to S1 were included in this study and were separated into five groups by age as follows: 6, 10, 14, 18, and 26 weeks. For each group mean was respectively 14.4 kg (± 1.2) (6 weeks group), 25.5 kg (± 1.0) (10 weeks group), 35.1 kg (± 1.4) (14 weeks group), 47.7 kg (± 1.8) (18 weeks groups) and 88.1 kg (± 1.6) (26 weeks group).

Pigs were positioned in dorsal recumbency as straight as possible. CT scans were performed on a multi-detector-row helical CT unit (General Electrics ^®^ BRIGHTSPEED 16 ELITE). The technical settings were 120 kV and 150 mA, and the pitch of 1 slice thickness was 0.625 mm. CT images were reconstructed using multi-planar reconstruction in the transverse and sagittal planes with a specific digital imaging software (Osirix ^®^). Transverse images were reconstructed parallel to the cranial endplate of the vertebral body, whereas the sagittal images were reconstructed at the midsagittal plane of the vertebra. The window width and level settings were standardised for all measurements (window width, 2000 Hounsfield units; window level, 500 Hounsfield units). All measurements were performed with this software.

**Fig 1:**
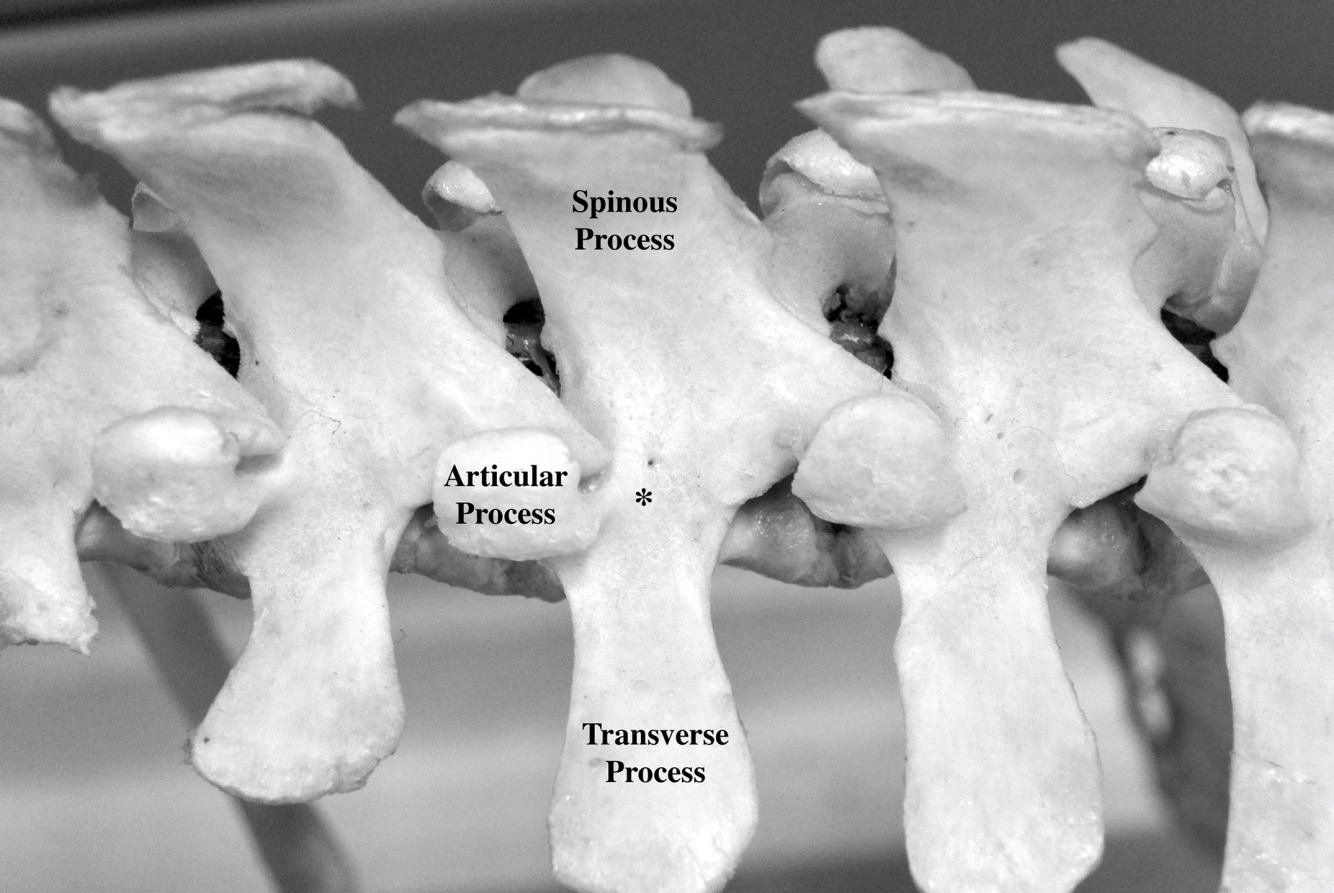
Anatomic specimen of the lumbar spine of a pig showing the entry point as described by McLain [12]. The entry point (*) was selected at the base of the cranial articular process of each vertebra immediately dorsal to the transverse process.

**Fig 2:**
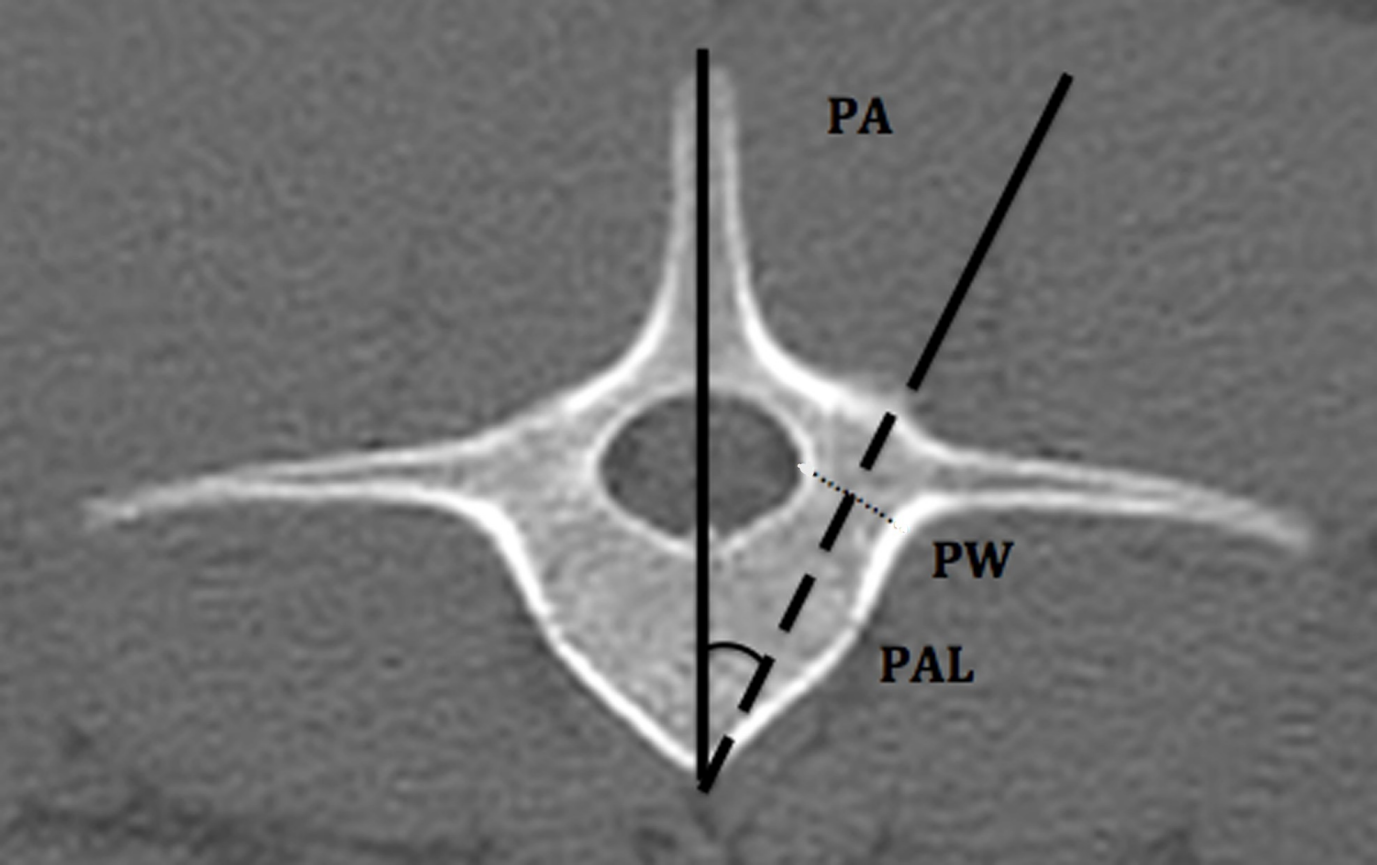
Description of the pedicle measurement in a third lumbar vertebra of 10-week-old pigs. The pedicle width (PW) is defined as the narrowest part of the pedicle (dotted line). The pedicle axis length (PAL) is defined as the length of a line bisecting the PW line starting from the base of the pedicle to the end the vertebral body (dashed line). The pedicle angle (PA) is defined as the angle between the PAL line and a line transecting the spinous process and the ventral vertebral process (black angle).

The implantation corridor for the pedicular screw was determined with a dorsal approach of the spine. As described by McLain [12], the entry point was selected at the base of the cranial articular process of each vertebra immediately dorsal to the transverse process (Fig 1). For each pedicle, three measurements (PW, PL, PAL) were performed on a transverse plane. The pedicle width (PW) was defined as the narrowest part of the pedicle and was measured from the inner cortex. The narrowest part was subjectively defined by observer for each pedicle. The pedicle axis length (PAL) was defined as the length of a line bisecting the PW line starting from the base of the pedicle to the end of the vertebral body. The pedicle angle (PA) was defined as the angle between the PAL line and a line transecting the spinous process and ventral vertebral process (Fig 2).

For each pig, the length of the thoracic spine (SL-T) and the length of lumbar spine (SL-L) was measured. The length of the thoracolumbar spine (SL-TL) was determined by the sum of the thoracic spine length and the lumbar spine length.

### Statistical analysis

For the assessment of the intra-observer reliability, five pigs were randomly selected and measurements were performed three times by the same observer for those pigs. An intraclass correlation coefficient (ICC) was then determined for the PA, PAL and PW. A non-parametric Wilcoxon test (p value<0.05) was used to compare each vertebra for the characteristics of the left and the right pedicles. With the same test, the PA angle was compared for each age group. Finally, a Mann-Witney test was used to compare the PAL and PW of thoracic and lumbar vertebrae pedicles. Statistical analyses were performed with a dedicated software program (XLSTAT ^®^).

## Results

CT scans from 21 pigs were used in this study. The numbers of pigs for each age group are reported in Table 1. A total of 452 vertebrae were analysed. All of the pigs got 6 lumbar vertebrae whereas 10 pigs got 15 thoracic vertebra and 11 pigs got 16 thoracic vertebrae. Therefore, a total of 326 thoracic vertebrae and 126 lumbar vertebrae were included in the study.

**Table 1:** Mean and standard deviation of the CT measurements of the pedicle. (PW = pedicle pedicle width, PAL = pedicle axis length, PA = pedicle angle).

	PIGLETS														
	6-week-old			10-week-old			14-week-old			18-week-old			26-week-old		
Weight (kg)	14.4 (± 1.2)			25.5 (± 1.0)			35.1 (± 1.4)			47.7 (± 1.8)			88.1 (± 1.6)		
SL-TL (cm)	38.7 (± 1.7)			46.9 (± 1.9)			54.8 (±0.8)			62 (±2.6)			74.2 (±3.1)		
SL-T (cm)	26.7 (± 1.1)			32.4 (±1.8)			37.9 (±1.2)			42.9 (±2.4)			51.2 (±1.8)		
SL-L (cm)	12 (± 0.6)			14.5 (±0.5)			16.9 (±0.9)			19.1 (±0.6)			23 (±1.5)		
	PA (°)	PAL (mm)	PW (°)	PA (°)	PAL (mm)	PW (°)	PA (°)	PAL (mm)	PW (°)	PA (°)	PAL (mm)	PW (°)	PA (°)	PAL (mm)	PW (°)
T1	30.7 ± 0.4	20.1 ±0.3	6.4 ±0.1	31.3 ±0.3	23.7 ±1.5	7.9 ±0.2	30.8 ±0.4	26.3 ±0.8	8.7 ±0.2	31.2 ±0.6	28.2 ±1.8	9.1 ±0.2	30.9 ±0.3	30.0 ±0.2	9.6 ±0.4
T2	20.6 ±0.5	20.7 ±0.5	5.9 ±0.1	21.0 ±0.2	24.3 ±1.6	7.5 ±0.2	21.2 ±0.8	27.0 ±0.9	8.3 ±0.2	20.8 ±0.6	28.8 ±2.1	8.7 ±0.1	21.1 ±0.3	30.4 ±0.2	9.1 ±0.4
T3-T10	15.4 ±0.8	22.7 ±0.9	5.3 ±0.3	15.3 ±0.9	26.4 ±2.0	6.7 ±0.4	15.2 ±0.8	28.8 ±0.7	7.6 ±0.3	15.2 ±0.7	30.2 ±2.0	8.1 ±0.4	15.2 ±0.7	32.1 ±0.6	8.4 ±0.4
T11	19.6 ±1.6	22.6 ±0.6	5.9 ±0.3	19.5 ±1.1	26.0 ±2.3	7.6 ±0.2	19.6 ±1.3	28.4 ±0.4	8.3 ±0.1	19.9 ±0.3	29.7 ±1.9	8.6 0.3	20.3 ±0.3	31.3 ±0.2	9.1 ±0.4
T12	28.5 ±0.4	22.1 ±0.6	6.3 ±0.2	28.9 ±0.5	25.8 ±2.1	7.9 ±0.2	28.7 ±0.3	28.0 ±0.3	8.5 ±0.1	29.2 ±0.4	29.3 ±1.8	8.9 ±0.2	28.7 ±0.4	31.0 ±0.2	9.3 ±0.4
T13-T16	31.9 ±1.0	21.3 ±0.6	6.9 ±0.3	31.9 ±0.8	24.9 ±1.3	8.3 ±0.3	31.5 ±0.9	27.4 ±0.4	9.0 ±0.2	31.3 ±0.9	29.1 ±1.9	9.3 ±0.3	32.0 ±0.6	30.6 ±0.5	9.8 ±0.4
L1-L4	32.5 ±1.9	23.1 ±0.6	6.6 ±0.3	32.3 ±1.4	26.6 ±0.7	8.0 ±0.3	32.1±0.7	28.6 ±0.5	8.7 ±0.3	32.3 ±0.3	29.9 ±2.0	9.1 ±0.4	32.8 ±0.4	31.7 ±0.5	9.5 ±0.3
L5	38.3 ±0.5	21.9 ±0.6	6.3 ±0.2	38.0 ±0.7	25.5 ±0.2	7.9 ±0.3	37.7 ±1.4	27.8 ±0.3	8.6 ±0.3	37.8 ±1.4	29.0 ±1.9	9.0 ±0.4	37.3 ±0.2	30.8 ±0.7	9.3 ±0.3
L6	41.9 ±1.3	21.0 ±0.4	6.7 ±0.2	41.7 ±0.4	24.9 ±0.3	8.1 ±0.3	42.4 ±1.1	27.2 ±0.4	8.8 ±0.3	42.5 ±2.4	28.4 ±2.1	9.3 ±0.4	42.4 ±0.6	29.9 ±0.8	9.6 ±0.3

**Fig 3:**
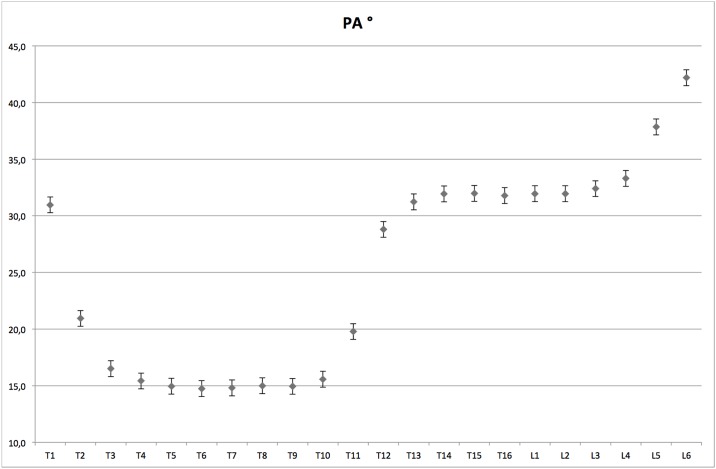
Evolution of the pedicle angle along the thoracolumbar spine of the pig. An abrupt modification of the PA between T10 and T11 vertebra was observed, which corresponds to the anticlinal vertebra, which is the vertebra for which the spinous process is nearly perpendicular to the vertebral body.

In Table 1, the mean and standard deviation of the CT measurements of the pedicle and spinal length are presented for the different ages. Pedicles are statistically larger but not longer for the lumbar vertebrae. An important variation of the PA is observed along the spine. In all pigs, an abrupt modification of the PA between T10 and T11 was observed. In pigs, the T11 vertebrae corresponds to the anticlinal vertebra (Fig 3) which is the vertebra for which the spinous process is nearly perpendicular to the vertebral body. No significant difference was found for the PAs of the different age groups. Therefore, the PAs did not change during the growth of the pigs.

The growth of the pedicle over time is reported in Table 2. The growth of the pedicle was maximal between the sixth and tenth weeks and then decreased slowly over time.

**Table 2:** Growth of the pedicle over time. (PW = pedicle pedicle width, PAL = pedicle axis length, PA = pedicle angle, SD = standard deviation).

			Pedicle Growth (mm/week)					
			Thoracic segment			Lumbar segment		
			Mean	SD	Min-Max	Mean	SD	Min-Max
***Growth period***	*6*^*th*^*-10*^*th*^ *week*	PAL	**0.90**	±0.06	0.84–0.97	**0.86**	±0.05	0.83–0.98
		PW	**0.38**	±0.02	0.32–0.43	**0.36**	±0.04	0.34–0.41
	*14*^*th*^*-10*^*th*^ *week*	PAL	**0.61**	±0.06	0.51–0.71	**0.53**	±0.05	0.46–0.61
		PW	**0.20**	±0.04	0.15–0.27	**0.16**	±0.02	0.14–0.18
	*14*^*th*^*-18*^*th*^ *week*	PAL	**0.39**	±0.05	0.34–0.49	**0.33**	±0.03	0.30–0.38
		PW	**0.11**	±0.02	0.08–0.13	**0.10**	±0.02	0.08–0.12
	*18*^*th*^*-26*^*th*^ *week*	PAL	**0.21**	±0.03	0.16–0.26	**0.22**	±0.02	0.19–0.24
		PW	**0.05**	±0.02	0.03–0.07	**0.05**	±0.01	0.04–0.06

No significant difference was observed between the left and right pedicles.

The ICC was 0.87 (0.80–0.94) for the PA, 0.86 (0.80–0.89) for the PAL and 0.89 (0.81–0.94) for the PW.

## Discussion

Pigs are one of the most representative animal models for spinal research, particularly for scoliosis [4, 9, 10, 12]. The implantation of pedicular screws is particularly difficult in young pigs because of their size and the quality of the bone. Morphometric data on pig pedicles are important to improving the planning of experimental studies and facilitating surgery using pedicular screws. This phase of planning is important for preventing perioperative accidents and limiting postoperative complications. From an ethical viewpoint, such planning reduces the use of live animals during an experimental study, which clearly complies with the current recommendation in experimental studies known as the rules of the 3Rs. [13]. Therefore, the present study provides valuable CT reference values for the safe implantation corridors for pedicular screws in the porcine thoracolumbar spine.

In humans, the pedicular screw is a commonly used surgical procedure to correct spinal deformity or instability caused by scoliosis, tumours or fractures. A pedicular screw with connecting rods provides the secure stabilisation of all 3 columns of the spine. A pedicle screw placed perfectly in the vertebral pedicle has better contact with the cortical bone and stronger fixation. The vertebral pedicle also represents a stronger site for screw placement than the vertebral body because the pedicle trabeculae and cortex are thicker and stronger than those in the vertebral body [14–17]. The pig is a well-accepted model for research on scoliosis, and pedicular screws are frequently used in these studies [4, 6–8, 10, 18]. The main factors that influence primary pedicular screw stability are the size of the screw, position of the screw and bone density [19]. Although bone density is not under the surgeon's control, the size and position of the screw are. Therefore, knowledge of pedicular anatomy is critical when using a pedicular screw.

The selected entry point of the pedicle is based not only on an anatomic description of the L4 vertebra [12] but also on the surgeon's personal experience of the insertion of a pedicle screw in the pig spine. This entry point is easily exposed and located via a dorsal approach. Thus, this entry point is a suitable site for the implantation of a pedicular screw.

Several other studies have been performed on porcine spine morphometry [12, 20]. However, Busschner reported only on the morphometry of 4-month-old pigs [20] and McLain described only the L4 vertebra of 60 kg pigs [12]. To our knowledge, this is the first report of the pedicle implantation corridor in very young pigs for all thoracolumbar vertebrae. Information about the pedicle anatomy young pigs is of interest because several studies have reported the use of very young pigs to benefit from the growing potential of the pigs. For example, Odent [7] and Fekete [7, 18] used pigs between 4 and 6 weeks of age.

A comparison of our results with the literature indicates that a similar size and angulation were found for pigs of a similar age. As described by Busschner and McLain, we found that between 4 and 5 months old, the pig pedicle width is similar to that of the human pedicle. Therefore, pigs are an interesting model for studies on pedicle screws [12, 20] and spinal fixation with pedicle screws.

Based on current recommendations, the ideal pedicle screw size should be 80% or less than the size of the pedicle [15, 16]. Therefore, the current study should be used as a guide to select the correct screw size when using pigs as a model for transpedicular fixation research. For example, pedicular screws used in the lumbar vertebra of 10-week-old pigs should not be larger than 6.5 mm; however, in paediatric patients, oversized screws up to 115% of the inside pedicle diameter may be used without causing a significant decrease in the holding power of the screw because of the plasticity of the pedicular cortex [15, 16].

Notably, the PW increased from the cranial to caudal axis. Therefore, the pedicular screw size may be larger in lumbar vertebrae than thoracic vertebrae, and the holding power of screws in the lumbar vertebrae would then be superior to those in the thoracic vertebrae. These conclusions are consistent with a recent study on screw pull-out strength in pigs, which demonstrated that a pedicular screw in the lumbar vertebrae (L1 to L5) has better holding power than a screw in the thoracic vertebrae (T5-T9) [21].

To our knowledge, this is the first report of pedicle characteristics in pigs. Information about these pedicle dimensions is of interest for the correct selection of screw length. In humans, the recommended pedicle screw length is no more than 70% of the length of the pedicle to avoid perforation of the transcortex of the vertebrae by the screws and injury to vital structures [15–17]. However, Le Cann showed that in immature pigs, a bicortical pedicle screw is stronger than a monocortical screw [21]. When working on young piglets, a screw length of a least the PAL should be used if bicortical anchorage is required by the surgeon.

As in humans, the pedicle angle changes along the porcine spine (Fig 2). We found comparable pedicle angles to those reported in the literature [12, 20]. The pedicle angle does not change during growth of the spine. It is important to respect the pedicle angle during screw insertion to avoid misplacement of the screw. Poor screw positioning may lead to spinal violation and potentially severe neurological lesion if the screw insertion angle is more oblique than the pedicle angle. By contrast, if the screw insertion angle is straighter, this could lead to poor bone anchoring, which may lead to screw loosening during the post-operative period. The pedicle angle reported in this study is for the optimal insertion angle of the screw. However, because the screw diameter is smaller than the pedicle width, a few degrees of variation of the insertion angle are acceptable.

In humans, Roy-Camille recommended that the pedicle screw be inserted in a straight (vertical) direction [22, 23]. Others propose that a more oblique trajectory is safer and allows for a better bone purchase [24, 25]. However, a biomechanical study shows that straight screw insertion in the lumbar vertebrae results in a more stable pedicle-screw construct than the angle screw insertion technique [26]. As shown in our study, the pig pedicle angle is more oblique than the human angle. Therefore, using a straight screw insertion technique could lead to a high misplacement rate in pigs and an oblique trajectory should be used. This conclusion was also reported for sheep [13].

In human being to get a correct bone cortical trajectory of the pedicle screw, the sagital or cephalad angle of insertion is important respect. In pigs, as the pedicle is not clearly define as in human being, the sagital angle of the pedicle corridor is not easily determined on pigs. Indeed, The screw is more more a vertebral body screw rather than a pedicle screw as in human. Thus, we beleive that a trajectory perpendicular to the sagital plane (sagital angle of 0°) is needed in pig.

There are several limitations of our study. First, different piglets were used in each group and there were few piglets per group; therefore, the growth of the pedicle should be considered with caution. However, because heavy selection pressure is applied in pig breeding, pigs from the same breeder are similar and few anatomic variations should be observed. In our study, all pigs were from the same breeder, and this study is thus a good approximation of pedicle growth. In the same way, only one breed of pigs were used in this study. Thus, transposition of those measurements to other breeds should be made with caution. Nevertheless we believe that those guidelines could be used in breeds close the landrace such as other Landrace pigs or Large White. Second, this study is not a direct anatomic study. Measurements were performed on CT scans, which may lead to some approximation in the measurements. This technique was selected because CT is a non-invasive imaging modality and is a well-accepted method to assess spinal and vertebral morphometry in vivo. CT has been widely used in both humans and animal models [13, 27, 28]. The CT scan parameters (slice thickness, pitch, and window width) used in this study follow the recommendations for orthopaedics studies and are consistent with published spinal CT imaging protocols. Third, as CT scan used in this study were not injected and some of them were performed on dead animal, we were not able to determine the distance between vital structures, such as aorta or caudal vena cava, and the vertebra. Thus we were not able to give any recommendation to avoid iatrogenic damage to those vessels when using bicortical screws. Finally, these corridors of implantation were only described in normal pigs. In scoliotic deformity models, several deformities of the spine are observed. These deformities may impact the pedicle morphometry and modify the implantation corridors described here. In humans, length asymmetry of the pedicle is reported in scoliotic vertebrae. The pedicle of the concave side is shorter and wider than that of the contralateral side [29, 30]. This asymmetry was not reported in a porcine scoliosis model, perhaps because in humans, pedicle asymmetry is only reported in severe scoliosis and appears later during the scoliosis deformation process. Interestingly, in a study on pedicle morphometry in scoliotic patients, the pedicle entry point and orientation relative to the vertebra were not affected by the deformities. However, as the position of the vertebra is highly affected by the spinal deformities, the pedicle orientation is changed along the spine, such that when the three-dimensional location of the vertebra is known, the entry point and pedicle screw orientation are the same as in a normal vertebra [29]. Similar modifications should be considered in a porcine scoliosis model, and this should be noted when using pedicle screws in scoliosis deformity models.

## Conclusions

In conclusion, this study provides a quantitative database of pedicle screw implantation corridors in pigs of different ages. When using pedicle screws in experimental studies in pigs, these results should be considered for selecting the most suitable implants for the study but also to ensure a correct and safer screw position. Improving study procedures may limit postoperative complications and pain, thereby limiting the use of live animals.
